# A Novel Freshwater to Marine Evolutionary Transition Revealed within *Methylophilaceae* Bacteria from the Arctic Ocean

**DOI:** 10.1128/mBio.01306-21

**Published:** 2021-06-22

**Authors:** Arthi Ramachandran, Susan McLatchie, David A. Walsh

**Affiliations:** a Department of Biology, Concordia University, Montreal, Quebec, Canada; CEH-Oxford

**Keywords:** climate change, marine microbiology, metagenomics, methanol, genome evolution

## Abstract

Bacteria inhabiting polar oceans, particularly the Arctic Ocean, are less studied than those at lower latitudes. Discovering bacterial adaptations to Arctic Ocean conditions is essential for understanding responses to the accelerated environmental changes occurring in the North. The *Methylophilaceae* are emerging as a model for investigating the genomic basis of habitat adaptation, because related lineages are widely distributed across both freshwater and marine ecosystems. Here, we investigated *Methylophilaceae* diversity in the salinity-stratified surface waters of the Canada Basin, Arctic Ocean. In addition to a diversity of marine OM43 lineages, we report on the genomic characteristics and evolution of a previously undescribed *Methylophilaceae* clade (BS01) common to polar surface waters yet related to freshwater sediment *Methylotenera* species. BS01 is restricted to the lower-salinity surface waters, while OM43 is found throughout the halocline. An acidic proteome supports a marine lifestyle for BS01, but gene content shows increased metabolic versatility compared to OM43 and evidence for ongoing genome-streamlining. Phylogenetic reconstruction shows that BS01 colonized the pelagic ocean independently of OM43 via convergent evolution. Salinity adaptation and differences in one-carbon and nitrogen metabolism may play a role in niche differentiation between BS01 and OM43. In particular, urea utilization by BS01 is predicted to provide an ecological advantage over OM43 given the limited amount of inorganic nitrogen in the Canada Basin. These observations provide further evidence that the Arctic Ocean is inhabited by distinct bacterial groups and that at least one group (BS01) evolved via a freshwater to marine environmental transition.

## INTRODUCTION

Studies in low-latitude oceans have provided numerous insights into the eco-evolutionary processes that underlie patterns of marine bacterial biogeography (1–3). However, bacterial communities inhabiting polar oceans, particularly the Arctic Ocean, are far less studied. There are a number of characteristics of the Arctic Ocean that make this region informative to include in studies on bacterial biogeography and evolutionary diversification in the global ocean. The Arctic Ocean is uniquely surrounded by Eurasian and North American land masses, which restrict water exchange with other oceans and influence ocean hydrology through freshwater input via large river systems (4–7). A second motivation is that increasing global temperatures are having a profound influence on the Arctic. In addition to warming (8, 9), the Arctic Ocean is freshening due to a dramatic loss of sea ice, increased precipitation, and river discharge (6, 7). The assembly of distinct bacterial communities in the Arctic Ocean in response to this unique and changing environmental setting is evident (10–13), as is the existence of Arctic-adapted ecotypes within globally prevalent marine bacteria such as SAR11 and SAR202 (14, 15). Moreover, time-series studies in the Canada Basin (Western Arctic) have shown the subsequent increase in stratification due to surface freshening, which affects nutrient transport and primary production in the photic zone (16, 17). There is evidence that the physicochemical changes are influencing microbial community structure. For example, a study comparing microbial community structure in the Beaufort Sea before and after the 2007 record sea ice minimum demonstrated significant differences in all three domains of life (18). In combination, such studies are beginning to reveal the unique community composition and genomic adaptations within Arctic marine microbiomes. Further studies that compare Arctic populations with their lower-latitude relatives should broaden our understanding of how bacterial taxa are adapted to life in the Arctic Ocean and provide insights into how these communities may respond to the rapid environmental changes underway.

The *Methylophilaceae* are emerging as a model for investigating evolutionary diversification and habitat adaptation in aquatic ecosystems, as closely related lineages are distributed across marine and freshwater ecosystems. *Methylophilaceae* are methylotrophs specialized to use one-carbon (C1) compounds, particularly methanol (19–21). Evolutionary studies based on comparative genomics suggest that ancestral *Methylophilaceae* inhabited sediments and subsequently colonized freshwater pelagic habitats (origin of LD28 and PRD01a001B clades) before further diversifying into marine pelagic habitats (OM43 clade) (22, 23). The transition from a sediment to a pelagic lifestyle involved extensive genome reduction, while the transition from freshwater to marine habitats involved metabolic innovation via lateral gene transfer (LGT) (23).

Within the *Methylophilaceae*, the marine OM43 lineage is among the most successful bacterial groups in the ocean, inhabiting diverse environments from tropic to polar seas (19, 23–29). OM43 is commonly found in coastal waters and brackish environments (19, 24, 26–28) and is often associated with phytoplankton blooms (29, 30). Phylogenetic analyses using 16S-23S internal transcribed spacer (ITS) sequences shows that OM43 is broadly divided into two ecotypic clusters, OM43-A (represented by strain HTCC2181) and OM43-B (also referred to as Hawaii-Red Sea [H-RS] cluster) (28). OM43-B is associated with low-chlorophyll *a* and/or warm oceans, whereas OM43-A is more prevalent in colder, higher-productivity waters (28). Additional OM43 microdiversity exists (e.g., OM43-A1 and OM43-A2) that may reflect further niche specialization. Given the broad distribution of *Methylophilaceae* in freshwater to marine habitats and their diversification linked to differences in salinity, temperature, and primary productivity, these methylotrophs may be an informative group for investigating bacterial adaptation in the rapidly changing Arctic Ocean.

In this study, we characterized the phylogenetic and genomic diversity of *Methylophilaceae* in the Canada Basin, Arctic Ocean. A major feature of the Canada Basin is the Beaufort Gyre. As of 2018, the freshwater content of the Canada Basin has increased approximately 40% relative to the 1970s because of increased sea ice melt and river water accumulation driven by an anticyclonic Beaufort Gyre (7). Stratification is increasing, and nutrient availability and primary production are shifting as a result of this freshening (8, 16, 17, 31, 32). Here, we provide a snapshot of *Methylophilaceae* diversity in vertically stratified metagenomes located along a latitudinal gradient of the Canada Basin. In doing so, we report on the discovery, genomic characteristics, and evolutionary origin of a previously undescribed lineage of marine *Methylophilaceae* that appears to be common in polar oceans.

## RESULTS

### Environmental context.

*Methylophilaceae* diversity was investigated along a four-station (CB2, CB4, CB8, and CB11) latitudinal transect (∼73° to 77°N) at 150^o^W in the Canada Basin during late summer-autumn of 2015 (Table 1; also see Fig. S1 in the supplemental material). The summer mixed layer depth ranged between 10 and 30 m. Surface (5 to 7 m) salinity ranged from 25.7 to 27.3 PSU, and nitrate concentrations were below the detection limit. The deep chlorophyll maximum (DCM) was located between 25 and 79 m, where salinity ranged from 29.7 to 31.5 PSU. In the deeper Pacific winter waters (PWW; defined as a salinity of 33.1 PSU), nitrate concentration was approximately 16 mmol/m^3^.

10.1128/mBio.01306-21.1 FIG S1 Map of the study location in the Canada Basin, Arctic Ocean. Download FIG S1, EPS file, 1.6 MB. Copyright © 2021 Ramachandran et al. 2021 Ramachandran et al. https://creativecommons.org/licenses/by/4.0/ This content is distributed under the terms of the Creative Commons Attribution 4.0 International license.

**TABLE 1:** Location and environmental characteristics of samples collected for metagenomic analyses

Station	Latitude (N)	Longitude (W)	SML^*a*^ depth (m)	Sample^*b*^ feature	Depth (m)	Temp (°C)	Salinity (PSU)	Fluorescence (mg/m³)	Nitrate (mmol/m³)	Silicate (mmol/m³)	Phosphate (mmol/m³)
CB2	72°59′	149°59′	10	Surface	6.3	−1.26	25.7	0.15	bdl^*c*^	2.55	0.52
				DCM	70.7	−0.89	31.5	0.33	4.45	10.05	1.07
				PWW	181.3	−1.45	33.2	0.05	15.98	32.9	1.84
CB4	75°00′	150°00′	30	Surface	4.5	−1.389	26.1	0.12	bdl	2.43	0.51
				DCM	80	−0.03	31.2	0.23	4.65	10.8	1.11
				PWW	212.3	−1.47	33.1	0.05	16.13	33.7	1.87
CB8	76°59′	149°58′	16	Surface	6	−1.46	27.2	0.19	bdl	2.73	0.54
				DCM	61.7	−0.15	31	0.3	0.35	5.13	0.78
				PWW	217.8	−1.45	33.1	0.05	16.24	35.02	1.9
CB11	78°59′	149°59′	17	Surface	7.5	−1.48	27.3	0.24	bdl	2.83	0.56
				DCM	27.6	−1.04	29.7	0.25	bdl	3.01	0.64
				PWW	194.6	−1.46	33.2	0.05	15.82	35.04	1.91

a SML, surface mixed layer.

b DCM, deep chlorophyll maximum; PWW, Pacific winter water.

c bdl, below detection limit.

### *Methylophilaceae* in the Canada Basin.

**FIG 1:**
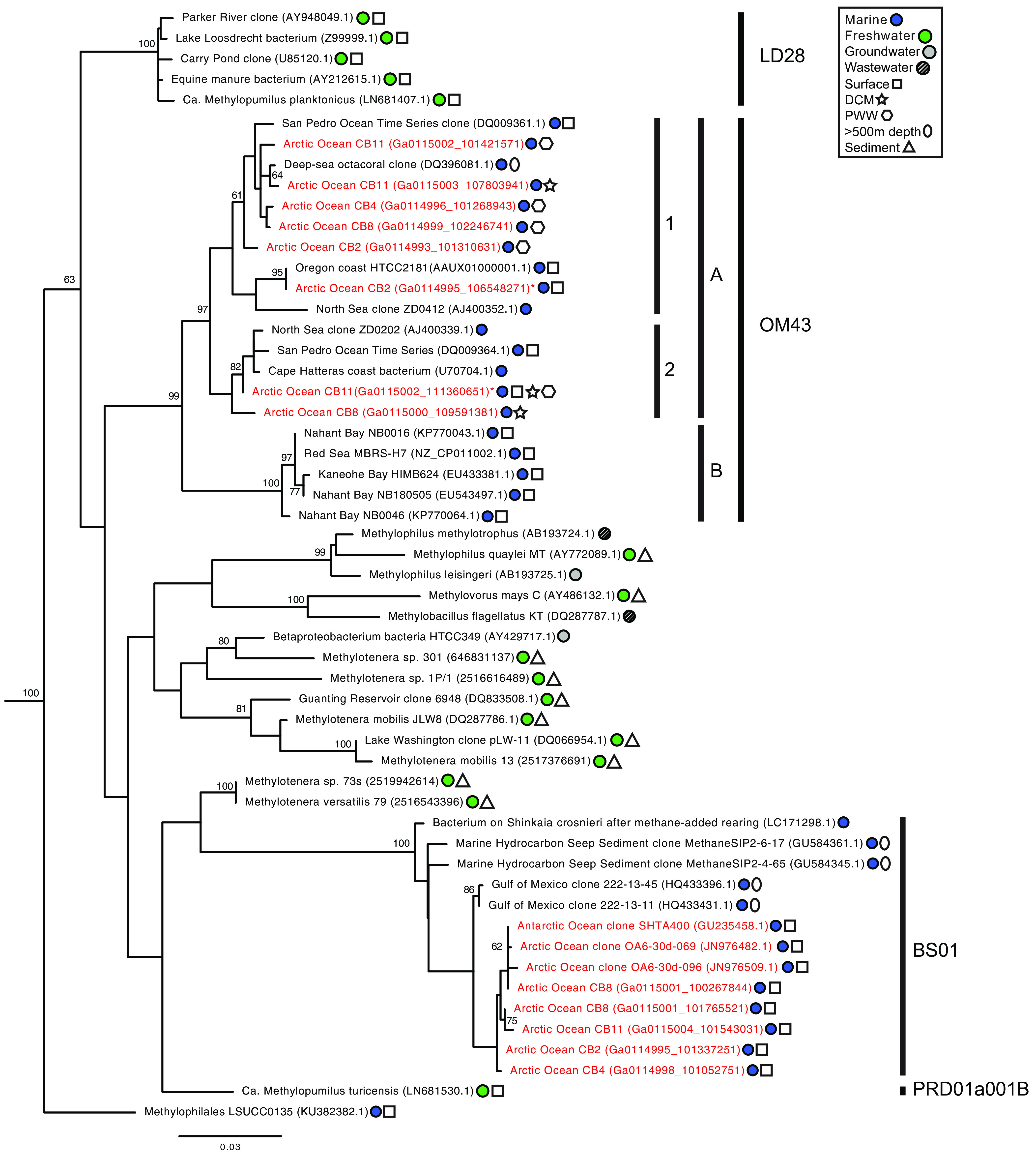
Phylogenetic analysis of 16S rRNA genes from *Methylophilaceae* from Canada Basin metagenomes and a diversity of aquatic ecosystems. The tree was inferred using maximum likelihood (500 bootstraps) and GTR + gamma distribution (four categories) with invariant site model of evolution and the nearest-neighbor interchange heuristic search method. The tree was rooted using *Methylibium* as an outgroup to the *Methylophilaceae*. Sequences from the current study are highlighted in red. Only bootstrap values of >60 are included in the tree.

*Methylophilaceae* 16S rRNA sequences were analyzed in Canada Basin metagenome assemblies from surface, DCM, and PWW samples. Within OM43, 16S rRNA sequences from OM43-A1 and OM43-A2 were detected, while OM43-B was not (Fig. 1). We also identified 16S rRNA sequences distantly related to previously described marine (OM43) and freshwater (LD28 and PRD001a001B) *Methylophilaceae* in all surface water metagenomes. These sequences formed a clade (here referred to as BS01) with sequences previously recovered from Arctic and Antarctic surface seawater and bottom waters of the Gulf of Mexico (Fig. 1).

**FIG 2:**
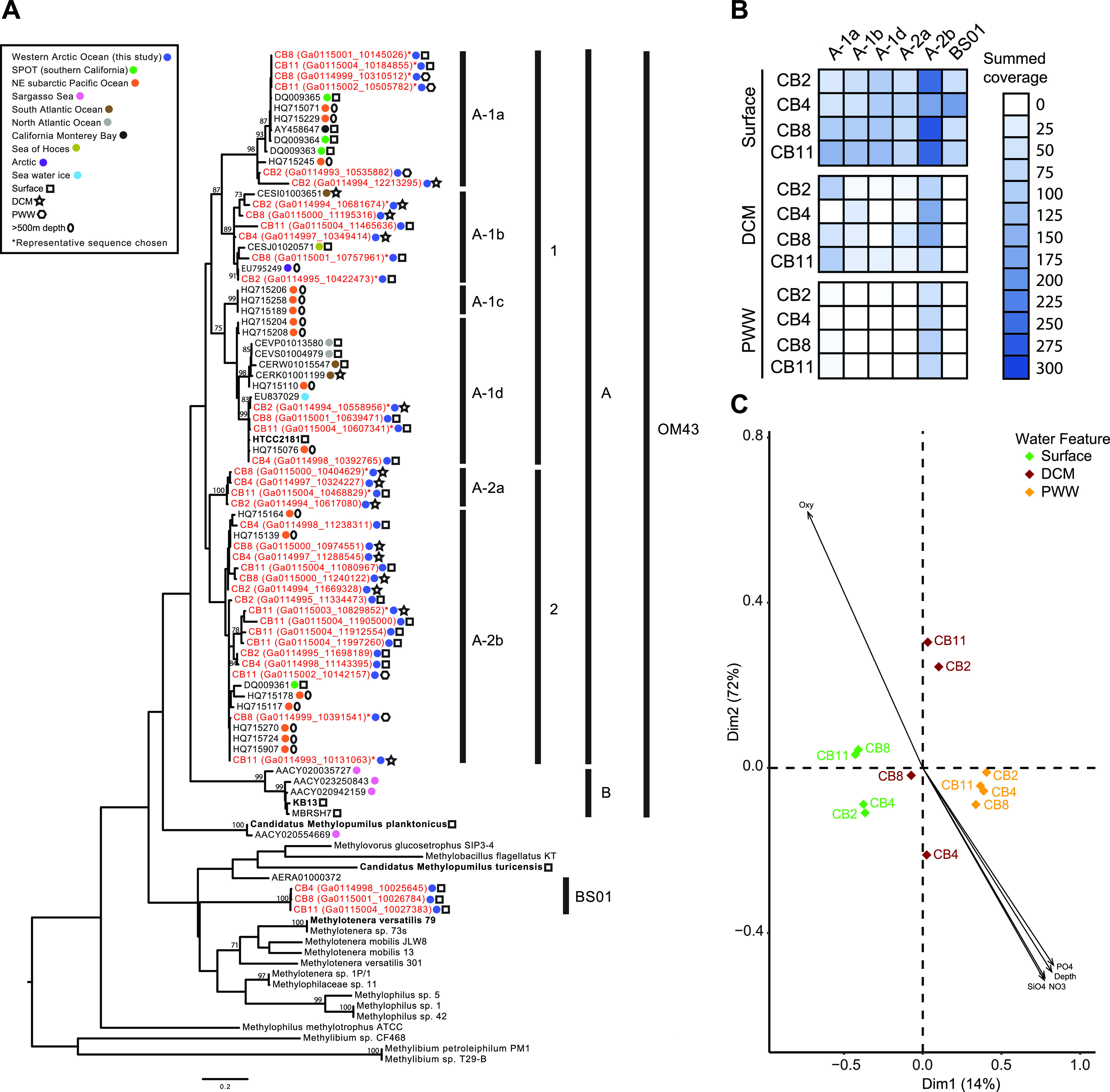
Diversity and biogeography of *Methylophilaceae* based on ITS variants recovered from Canada Basin metagenomes. (A) Phylogenetic analysis of the *Methylophilaceae* group across various aquatic regions and depths using the ITS region. The tree was inferred using maximum likelihood (500 bootstraps) and a GTR + gamma distribution (four categories) with invariants sites model of evolution and the nearest-neighbor interchange heuristic search method. Sequences from the current study are highlighted in red. Only bootstrap values of >60 are included in the tree. (B) Abundance of six ITS variants based on summed coverage in metagenome assemblies. (C) Principal coordinate analysis ordination of Bray-Curtis dissimilarities of Arctic samples based on summed coverage of six ITS variants.

We analyzed ITS diversity to provide finer phylogenetic resolution of *Methylophilaceae*. Thirty-nine ITS variants formed six ITS subclades (we designated these clades OM43-A1a to OM43-A1d and OM43-A2a to OM43-A2b) (Fig. 2A). Similar to 16S rRNA diversity, we detected ITS sequences from OM43-A throughout the water column as well as a distantly related group that likely represents BS01. OM43-A2b was detected in all Canada Basin metagenomes, irrespective of water layer, while OM43-A1 ITS clades (A1a, A1b, and A1d) were more restricted to the surface and DCM layers (Fig. 2B). A principal component analysis showed that most of the variation in OM43 diversity was in ITS subclade contribution to DCM assemblages, although the pattern was not related to any clear differences in environmental conditions or nutrient availability (Fig. 2C). Overall, 16S rRNA and ITS diversity demonstrated a diverse assemblage of OM43 bacteria in the upper layers of the Arctic Ocean and identified a previously undescribed lineage of *Methylophilaceae* in the ocean.

### Genomic characteristics of BS01.

**FIG 3:**
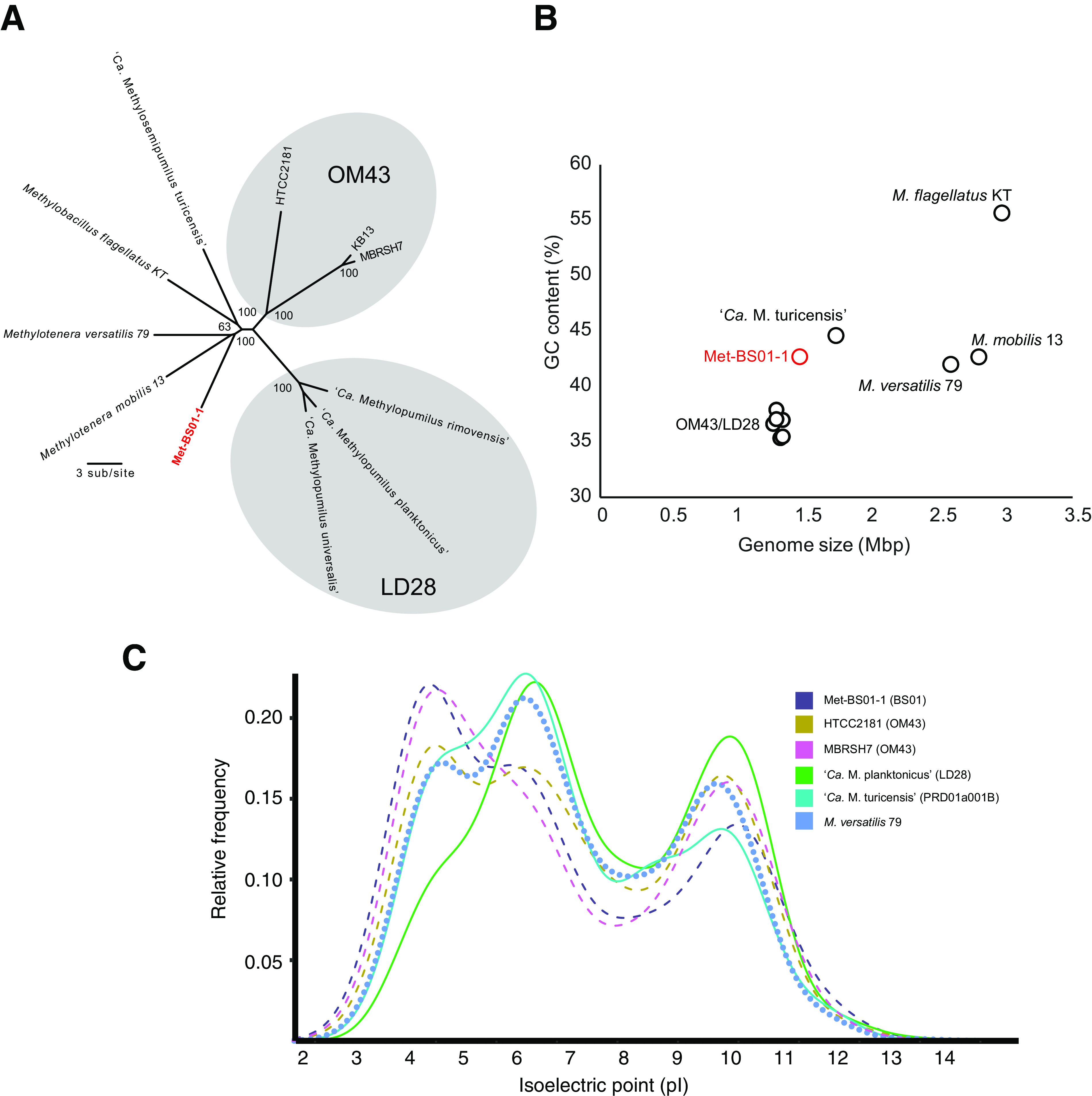
Phylogenomic comparison of BS01 with representative *Methylophilaceae* genomes. (A) Maximum likelihood phylogenetic analysis of a concatenated alignment of 48 orthologs shared between all *Methylophilaceae* genomes. Values at the nodes are bootstrap values (100 pseudoreplicates). (B) Scatterplot comparing G+C content and genome size. (C) Whole-proteome pI values versus relative frequency in select *Methylophilaceae* genomes from freshwater and marine habitats.

To further characterize BS01, we reconstructed a representative metagenome-assembled genome (MAG) from a CB2 surface water metagenome. The BS01 MAG (Met-BS01-1) was 1.48 Mb in length and 92% complete. Concatenated protein phylogeny showed Met-BS01-1 was more closely related to sediment-derived *Methylotenera* species than pelagic marine (OM43) or fresh (LD28 and PRD01a001B) water *Methylophilaceae* (Fig. 3A). In agreement with the phylogeny, Met-BS01-1 exhibited higher average amino acid identity (%) with genomes from freshwater *Methylotenera* and “*Candidatus* Methylosemipumilus turicensis” (62 to 68%) than marine OM43 genomes (53 to 55%) (Table S1). The Met-BS01-1 genome was more similar in size to those of the genome-streamlined pelagic OM43 and LD28 than *Methylotenera* strains (Fig. 3B, Table S1). However, GC content of Met-BS01-1 (43% G+C) showed the opposite trend, exhibiting higher similarity to *Methylotenera* than OM43/LD28 genomes (Fig. 3B). Previous studies on genome streamlining have reported shifts in amino acid usage as a response to nitrogen limitation, measured as increases in the lysine-to-arginine ratio of the proteome (23, 33, 34). The lysine (6%) and arginine (4.5%) content of the Met-BS-01 proteome is more similar to *Methylotenera* than OM43/LD28 genomes (Table S1). In total, these observations suggest that genome streamlining has occurred during the evolution of BS01 but that the commonly associated shift toward lower GC content and reduced nitrogen amino acid usage were not apparent.

10.1128/mBio.01306-21.5 TABLE S1 Characteristics of *Methylophilaceae* genomes. Download Table S1, PDF file, 0.03 MB. Copyright © 2021 Ramachandran et al. 2021 Ramachandran et al. https://creativecommons.org/licenses/by/4.0/ This content is distributed under the terms of the Creative Commons Attribution 4.0 International license.

Proteome amino acid content is a valuable trait for predicting the preferred habitat of an organism, since marine bacteria exhibit more acidic values of protein isoelectric points (pI) than freshwater bacteria (35). To elucidate habitat adaptation in BS01, we compared global pI plots between Met-BS01-1 and related *Methylophilaceae*. Met-BS01-1 exhibited the highest peak at an acidic pI (∼4.5), which is similar to marine OM43 genomes (HTCC2181 and MBRSH7) (Fig. 3C). In contrast, freshwater *Methylophilaceae* exhibit a peak at ∼6.5. Interestingly, the Met-BS01-1 pI plot exhibits a frequency of highly acidic proteins (∼4.5) that was more similar to the OM43-B representative (MBRSH7) than OM43-A (HTCC2181). Overall, the acidic skew of proteins in Met-BS01-1 provide strong support that BS01 is a lineage of marine *Methylophilaceae* rather than a freshwater lineage introduced to the Canada Basin by river discharge.

### Gene content variation and marine adaptation in BS01.

Gene content was compared between MetBS01-1 and a set of freshwater and marine *Methylophilaceae* genomes (Fig. S2). In total, 503 Met-BS01-1 genes were conserved among the analyzed *Methylophilaceae* genomes, while 349 genes were unique to Met-BS01-1. In agreement with the phylogenetic affiliation of BS01 with sediment *Methylotenera*, an additional 259 genes were shared between Met-BS01-1, *M. versatilis* 79, and *M. mobilis* 13. Genes shared exclusively between MetBS01-1 and one or more marine OM43 genomes were not detected.

10.1128/mBio.01306-21.2 FIG S2 Comparative genomics of shared gene content among *Methylophilaceae* strains visualized using Anvi’o. Download FIG S2, SVG file, 2.5 MB. Copyright © 2021 Ramachandran et al. 2021 Ramachandran et al. https://creativecommons.org/licenses/by/4.0/ This content is distributed under the terms of the Creative Commons Attribution 4.0 International license.

We next interrogated Met-BS01-1 for genes that may be associated with a marine lifestyle, such as osmoregulation and ion metabolism. A H^+^-translocating NADH dehydrogenase (NDH) was present in Met-BS01-1 rather than the Na^+^-translocating NADH:quinone oxidoreductase (NQR) that is often associated with marine bacteria (Fig. S3) (22). We identified 196 genes that exhibit highest similarity to homologs from outside the *Methylophilaceae* family. Of these, none recognizably originated from typical marine bacteria. However, several were associated with sodium transport, including a Na^+^/melibiose symporter related to *Alphaproteobacteria* and a small-conductance mechanosensitive channel and calcium/sodium antiporter related to *Gammaproteobacteria* (Fig. S3). An additional set of genes associated with Na^+^ metabolism were shared between Met-BS01-1 and *Methylotenera*, including Na^+^/proline (*putP*), Na^+^/H^+^-dicarboxylate (*gltT*), alanine/glycine:cation (*agcS*), and neurotransmitter/Na^+^ symporters, as well as Na^+^/H^+^ (*nhaA*) and monovalent cation/H^+^ antiporters (Fig. S3).

10.1128/mBio.01306-21.3 FIG S3 Summary of the distribution of metabolism modules across *Methylophilaceae* genomes. Download FIG S3, EPS file, 2.4 MB. Copyright © 2021 Ramachandran et al. 2021 Ramachandran et al. https://creativecommons.org/licenses/by/4.0/ This content is distributed under the terms of the Creative Commons Attribution 4.0 International license.

### BS01 energy and nutrient metabolism.

**FIG 4:**
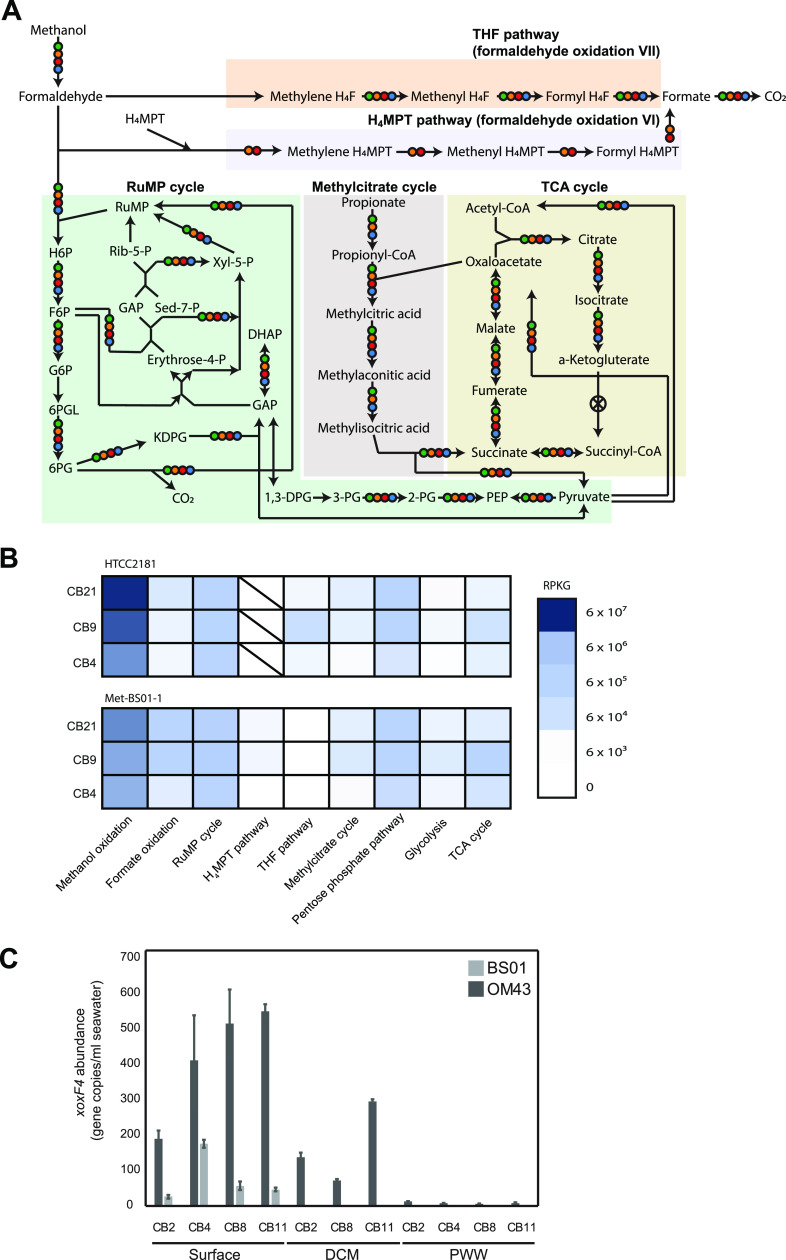
Reconstruction of methylotrophic metabolism in BS01 and comparison to other *Methylophilaceae*. (A) Distribution of central one-carbon metabolism genes. (B) Gene expression pattern for central carbon metabolism pathways in Canada Basin surface waters revealed through fragment recruitment of metatranscriptomics against Met-BS01-1 and HTCC2181 genomes. (C) Quantification of BS01 and OM43 abundances in the Canada Basin using qPCR analysis of *xoxF4* gene abundance. Error bars indicate standard deviation. DCM, deep chlorophyll maximum; PWW, Pacific winter water.

Metabolic reconstruction of Met-BS01-1 indicated the ability to grow on methanol as a sole source of carbon and energy (Fig. 4A). Similar to other pelagic *Methylophilaceae*, a single lanthanide-dependent methanol dehydrogenase (*xoxF4*) was present, while the calcium-dependent methanol dehydrogenase (*mxaF*) was not detected. Methylotrophic activity of BS01 in Arctic Ocean surface water was supported by an abundance of *xoxF4* transcripts in Canada Basin metatranscriptomes (Fig. 4B, Table S3). Quantitative PCR (qPCR) targeting either BS01 or OM43 *xoxF4* genes specifically verified that BS01 is restricted to the surface waters of the Canada Basin, while OM43 is more broadly present in the water column (Fig. 4C). Similar to other pelagic methylotrophs, Met-BS01-1 carried all genes for the tetrahydrofolate (H_4_F) pathway for formaldehyde oxidation, the ribulose monophosphate (RuMP) cycle for formaldehyde assimilation/oxidation, and formate oxidation via formate dehydrogenase (Fig. 4A). In addition, Met-BS01-1 possessed the tetrahydromethanopterin (H_4_MPT) pathways for formaldehyde oxidation, which was thought to be restricted to sediment methylotrophs (20) but recently identified in “*Ca.* M. turicensis” (23). Interestingly, we only detected Met-BS01-1 transcripts from the H_4_MPT pathway and not the H_4_F pathway in Canada Basin metatranscriptomes (Fig. 4B). Known genes involved in the processing of other C1 and C1-related compounds, including DMSP, glycine betaine, and methylated amines, were not present in Met-BS01-1.

10.1128/mBio.01306-21.7 TABLE S3 Gene expression pattern for central carbon metabolism genes in Canada Basin surface waters revealed through fragment recruitment of metatranscriptomics against Met-BS01-1 and HTCC2181 genomes. Download Table S3, XLSX file, 0.01 MB. Copyright © 2021 Ramachandran et al. 2021 Ramachandran et al. https://creativecommons.org/licenses/by/4.0/ This content is distributed under the terms of the Creative Commons Attribution 4.0 International license.

**FIG 5:**
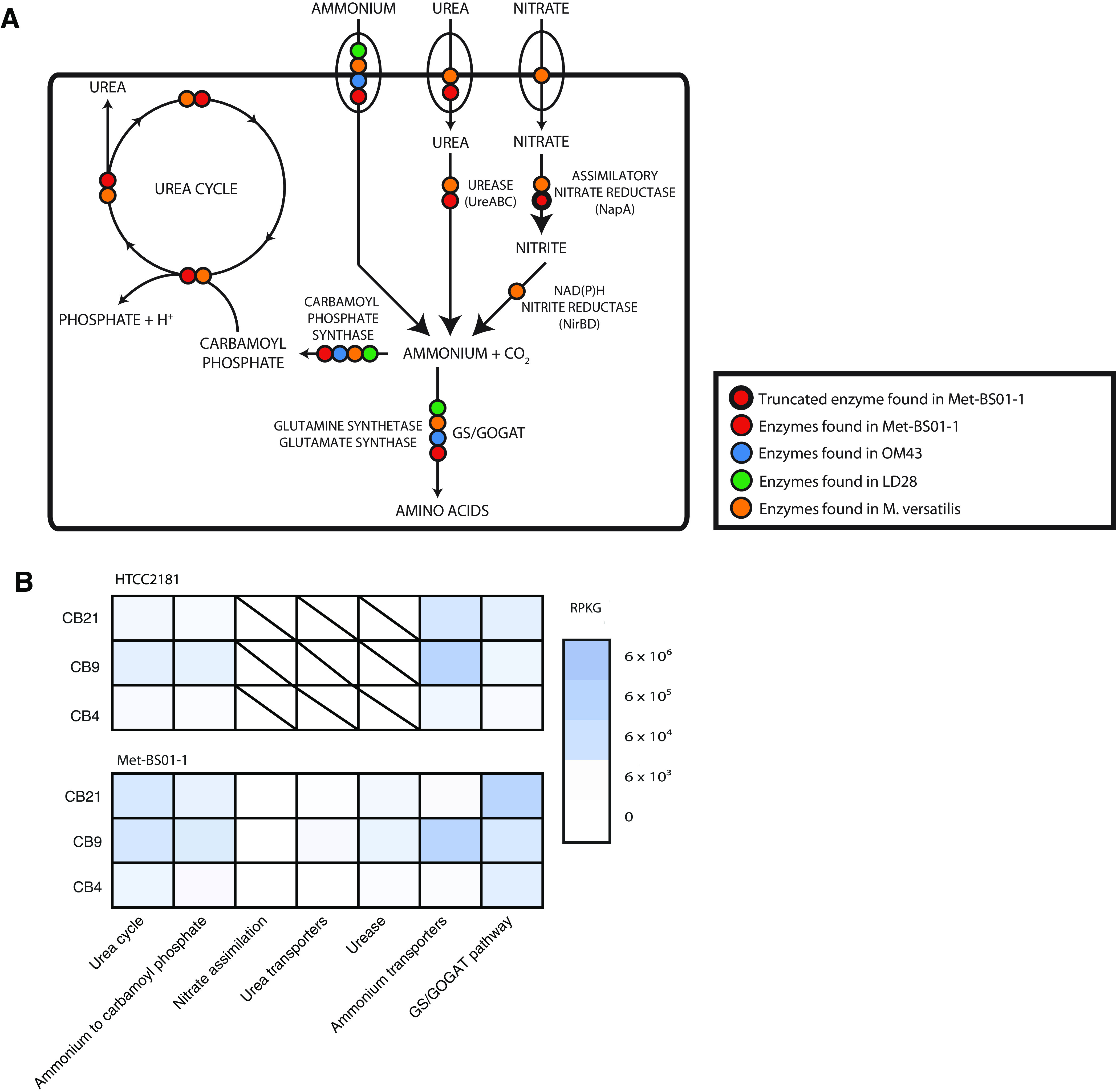
Reconstruction of nitrogen metabolism in BS01 and comparison to other *Methylophilaceae*. (A) Distribution of central nitrogen metabolism genes. (B) Gene expression pattern for central nitrogen metabolism pathways in Canada Basin surface waters revealed through fragment recruitment of metatranscriptomics against Met-BS01-1 and HTCC2181 genomes.

With respect to nitrogen acquisition, Met-BS01-1 encoded an ammonium transporter (*amtB*) and the glutamine synthetase/glutamine oxoglutarate aminotransferase (GS/GOGAT) assimilation pathway, similar to all known *Methylophilaceae* (Fig. 5A). The complete set of genes required for assimilatory nitrate reduction was not identified (missing nitrate and nitrite transporters and *nirB*), and *napA* was truncated, encoding only the last 385 amino acids of the typical 800+ amino acids, suggesting a nonfunctional pseudogene (Fig. 5A). In contrast to previously described pelagic methylotrophs, Met-BS01-1 shared the ability for urea utilization with *Methylotenera*. A urea ABC-type transporter encoded by the *urtABCDE* operon and an operon encoding the full urease enzyme and accessory proteins (*ureABCDEFG*) were present in Met-BS01-1 and exhibited highest similarity to orthologs from the freshwater *Methylotenera* sediment isolates (e.g., 80 to 93% for the UreA-UreC protein subunits). Urea use by BS01 was evident, as transcripts for urea transport and assimilation and the GS/GOGAT pathway were detected Canada Basin metatranscriptomics (Fig. 5B, Table S4). Met-BS01-1 also shared an incomplete urea cycle with the sediment methylotrophs. Genes annotated as amino acid transporters were not identified in Met-BS01-1. Interestingly, Met-BS01-1 encodes an ABC-type phosphate transport system as well as polyphosphate kinase and exopolyphosphatase, suggesting an ability to store phosphorus under nitrogen limited conditions and mobilize the stored phosphorus when enough nitrogen is available for growth.

10.1128/mBio.01306-21.8 TABLE S4 Gene expression pattern for central nitrogen metabolism genes in Canada Basin surface waters revealed through fragment recruitment of metatranscriptomics against Met-BS01-1 and HTCC2181 genomes. Download Table S4, XLSX file, 0.01 MB. Copyright © 2021 Ramachandran et al. 2021 Ramachandran et al. https://creativecommons.org/licenses/by/4.0/ This content is distributed under the terms of the Creative Commons Attribution 4.0 International license.

### BS01 biogeography across aquatic ecosystems.

**FIG 6:**
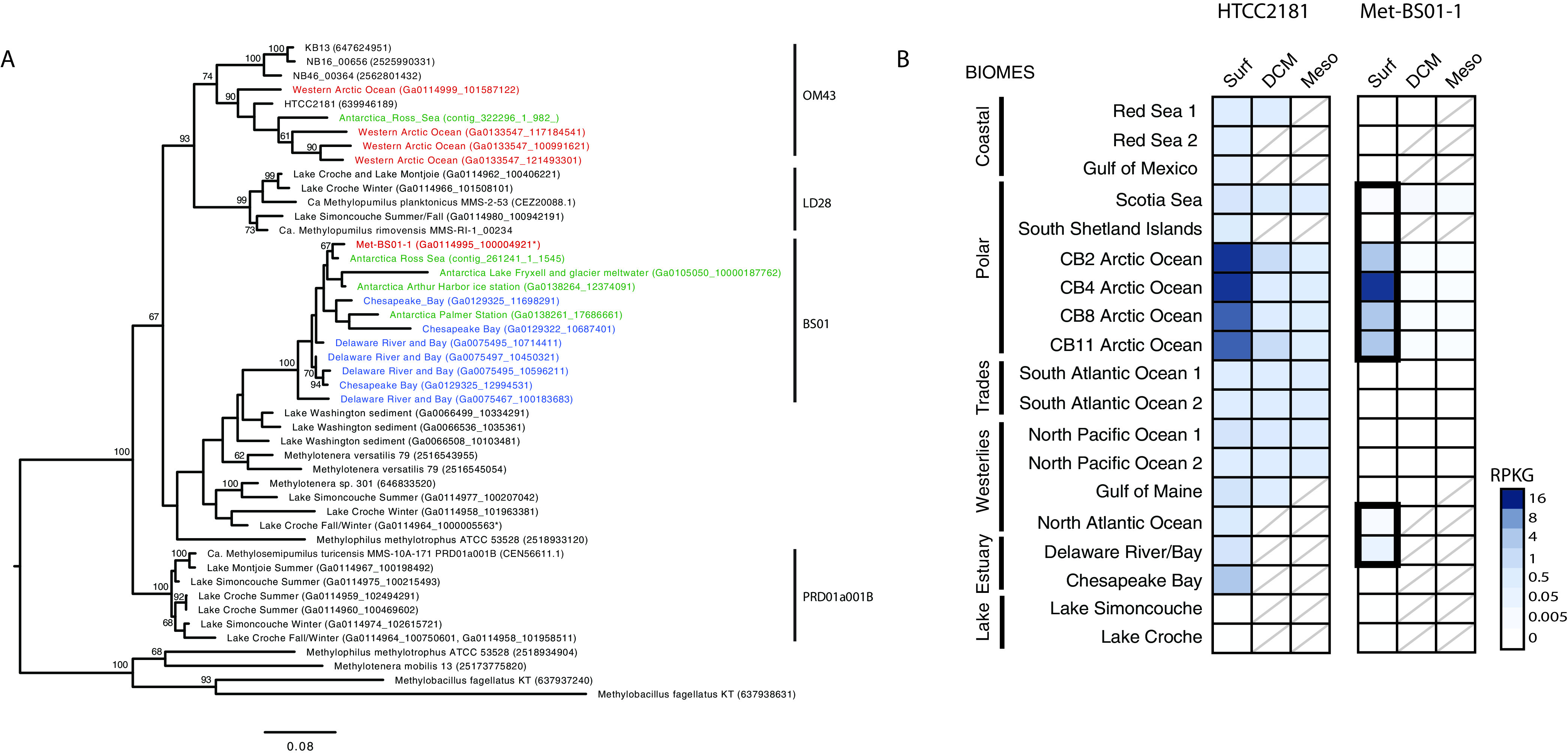
Biogeography of BS01 across the global ocean and estuaries. (A) Phylogenetic analysis of the *Methylophilaceae* family across multiple aquatic regions and depths using the XoxF4 methanol dehydrogenase protein recovered from metagenomes. The tree was inferred using maximum likelihood (500 bootstraps) and JTT + gamma distributed with invariants (four categories) sites model of evolution, with nearest-neighbor interchange heuristic search method. Colored sequences are those from the Arctic Ocean (red), the Antarctic (green), or estuaries (blue). Only bootstrap values of >60 are included in the tree. (B) Distribution of BS01 and OM43-A revealed through fragment recruitment of aquatic metagenomes against Met-BS01-1 and HTCC2181 genomes reported as reads per kilobase of the MAG per gigabase of metagenome (RPKG). The diagonal lines are to signify no metagenome data for that water column feature.

The presence of BS01 in ecosystems outside of the Arctic Ocean was investigated by applying a combination of phylogenetic marker (*xoxF4*) and fragment recruitment analyses to diverse aquatic metagenomes. We identified several BS01 *xoxF4* genes in metagenomes from Antarctic seawater and a broad *xoxF4* diversity in eastern North American estuary (Chesapeake and Delaware Bays) samples that ranged in salinity from 15 to 30 PSU (Fig. 6A). Since methanol dehydrogenase genes are prone to lateral gene transfer (36, 37), we verified the presence of BS01 in these samples using metagenomic fragment recruitment. Fragment recruitment only detected Met-BS01-1 in polar surface waters, including the Southern Ocean (Scotia Sea), and estuaries (Fig. 6B). In contrast to the restricted detection of Met-BS01-1, fragment recruitment against the HTCC2181 genome was observed for all marine biomes (coastal, polar, trades, and westerlies) analyzed as well as estuaries (Fig. 6B).

## DISCUSSION

### *Methylophilaceae* diversity in the salinity-stratified Arctic Ocean.

In this study, we discovered a diverse assemblage of *Methylophilaceae* in the salinity-stratified waters of the Canada Basin. Arctic *Methylophilaceae* were comprised of an array of marine OM43 subclades and a distantly related lineage, here termed BS01 (Fig. 1). All OM43 subclades identified in the Arctic Ocean were within OM43-A (Fig. 2A), in agreement with previous findings that OM43-A is generally more common in colder habitats than OM43-B (28). At lower latitudes, OM43-A is strongly associated with phytoplankton blooms (19, 25), which is a likely source of methanol for growth (38). In contrast, we find that OM43 was relatively rare in the chlorophyll maximum of the Canada Basin and was, in fact, more abundant in the extremely oligotrophic surface waters. This vertical structuring of OM43 was observed across all analyses, including the metagenome ITS diversity (Fig. 2B) and fragment recruitment analyses (Fig. 6B) as well as OM43 distribution assessed by quantitative PCR (qPCR) (Fig. 4C). One explanation for low abundance in the Canada Basin chlorophyll maximum is that it is comprised mostly of eukaryotic picophytoplankton (32), which may not be as significant a source of methanol as larger bloom-forming phytoplankton. What supports the methanol metabolism of the surface water *Methylophilaceae*? In addition to *in situ* production, methanol in the surface waters may originate from atmospheric deposition, which is elevated in the Arctic compared to lower-latitude regions due to the colder temperatures (39, 40). Another possibility is that Arctic *Methylophilaceae* are associated with phytoplankton blooms that periodically occur in the surface waters. In particular, under-ice blooms have been observed throughout the Arctic Ocean (41–45), including the Beaufort Sea (46, 47). Betaproteobacteria are present under the ice in the Beaufort Sea during the winter/spring (48, 49), and Collins et al. (77) identified OM43 under as well as within sea ice. Overall, these results demonstrate that marine *Methylophilaceae* are common in the stratified waters of the Canada Basin, and investigation of their seasonal dynamics would provide a deeper understanding of their ecological role in Arctic Ocean ecosystems.

### Freshwater-marine transitions within the *Methylophilaceae*.

Insights into the evolutionary adaptations associated with major habitat transitions, such as between marine and freshwater environments, can be revealed through comparison of closely related taxa from different habitats (22, 50, 51). Here, we provided strong evidence that BS01 represents a second lineage of marine *Methylophilaceae* that arose independently of marine OM43. The discovery of multiple evolutionary origins of pelagic marine *Methylophilaceae* provides a rare opportunity to compare the pathways of evolutionary adaptation to the ocean. OM43 is thought to have arisen from a sediment to pelagic transition in a freshwater ecosystem, followed by a second transition from fresh to marine waters (52). BS01 is related to freshwater sediment *Methylotenera* as well as pelagic *“Ca.* M. turicensis” and, therefore, may have originated through a sediment similar to pelagic and then a series of transitions from freshwater to marine. However, we propose that pelagic BS01 evolved along a different path, directly from a marine ancestor residing in sediments. Compared to freshwater *Methylotenera*, the BS01 proteome has undergone extensive changes in amino acid compositions (acidic shifts in pI) (Fig. 3C), which requires long evolutionary time (35). However, changes associated with oligotrophic conditions such as reduced GC content and a shift to less nitrogen-rich amino acids are not as striking. Although the Met-BS01-1 genome is approaching the small size of other oligotrophs (34), including LD28 and OM43, it is still considerably larger (Fig. 3B). Moreover, ongoing genome reduction was evident by the identification of partial deletion of the nitrate reductase, with only a *napA* pseudogene remaining. Hence, BS01 appears to have an established set of proteome modifications associated with a marine lifestyle but is at an intermediate stage with respect to pelagic adaptation. These findings further the notion that *Methylophilaceae* serve as a valuable model for “evolution in action” studies (52). Additional comparative studies that include new isolates (53) and MAGs from a broader diversity of environments, including marine sediments, should further advance our understanding of microbial habit transitions in aquatic ecosystems.

Lateral gene transfer plays a significant role in bacterial diversification, and the acquisition of genes involved in osmoregulation has been implicated in marine transitions (22, 54). Several sodium transporters were identified in the Met-BS01-1 genomes that may have originated by lateral transfer from *Gamma*- and *Alphaproteobacteria*. However, overall there was not a striking pattern of “marine gene” acquisition in BS01. Given that marine adaptation requires extensive adaptation across the whole proteome (Fig. 3C), we hypothesized that orthologous gene replacement of freshwater-adapted core proteins with more acidic marine homologs may have played a role in BS01 evolution. Phylogenetically related proteins that only differ slightly in their acidic amino acid content would have the most chance of successful incorporation in the recipient genome (35). With this in mind, we looked for evidence of OM43 serving as a donor of marine orthologs of core genes to BS01. We did not detect any genes exclusive to BS01/OM43. Moreover, BS01 genes shared with OM43 were consistently more similar to *Methylotenera* homologs, which is evidence against orthologous replacement. These results show that although BS01 and OM43 share the same habitat and are phylogenetically related, they apparently rarely undergo genetic exchange. A similar observation was made for a newly discovered freshwater lineage of SAR11 in ancient Lake Baikal, where Cabello-Yeves et al. wondered why the lineage had not acquired genetic material from previously established freshwater SAR11 relatives (55). Perhaps finer-resolution studies will identify orthologous replacements or even within-gene recombination events, as evolving aquatic bacteria share the same pool of marine or freshwater alleles.

### Niche differentiation of BS01 and OM43.

The coexistence of BS01 and OM43 in Canada Basin surface waters leads to questions of how these related methylotrophs are ecologically differentiated and how BS01 successfully competes with more established OM43 bacteria. Salinity may play a direct role in niche differentiation of OM43 and BS01, but neither the biogeographic distributions nor genome characteristics provide immediate insights about how. Both groups were detected across a broad salinity range in the Canada Basin and estuaries (Fig. 6B) and possessed similarly acidic proteomes (Fig. 3C). Both groups also appear to be highly specialized for methanol oxidation, but differences were apparent that may be ecologically relevant. Genome reduction in both groups has converged on a similar methylotrophic metabolism comprised of the same core metabolic modules identified by Salcher et al. (52): methanol oxidation via XoxF, formaldehyde oxidation via H_4_F and the RuMP cycle, and formate oxidation. However, one metabolic difference between BS01 and OM43 may be related to formaldehyde oxidation rates, because BS01 has retained the H_4_MPT pathway for formaldehyde oxidation. Evidently, the H_4_MPT but not the H_4_F pathway is expressed in the Canada Basin. Perhaps the H_4_MPT pathway can more rapidly remove toxic formaldehyde. Under conditions where growth is limited by inorganic nutrient availability and the reducing power of methanol is being funneled into maintenance energy generation rather than carbon assimilation and growth, perhaps the H_4_MPT pathway provides an advantage over the H_4_F pathway.

Another ecologically relevant difference between OM43 and BS01 may be related to nitrogen metabolism. Both groups have independently lost the genes for nitrate transport and assimilatory reduction while retaining ammonia transporters (see Fig. S3 in the supplemental material). However, although BS01 has undergone genome reduction compared to sediment dwellers, the full complement of genes for urea transport and utilization as a nitrogen source has been retained. Urea is present at relatively high but variable concentrations in the Arctic Ocean (56) and often exceeds that of ammonia. In fact, urea has been show to fuel Arctic nitrification (57). Sources of urea include riverine input, excretion, and sloppy feeding by zooplankton (58) and inputs from the melting of seasonal fast ice (59). Urea is hypothesized to replace ammonia as a waste product for organisms residing in confided habitats like sea ice because it is less toxic. The input from melting sea ice may be particularly relevant if these methylotrophs are associated with under-ice and ice-associated phytoplankton. The unique ability of BS01 to use urea under these conditions would provide an advantage over OM43 given the limited amounts of inorganic nitrogen in the Canada Basin. In fact, a seasonal study in the Arctic using stable isotope probing (SIP) showed no evidence of ^15^N urea uptake in summer, whereas winter samples yielded estimates of 30% isotopic labeling of bacterial populations, including *Betaproteobacteria* (60). Taken together, salinity, differences in C1 metabolism, and nitrogen utilization may all play a role in niche differentiation. Although genomes can provide some insights into metabolic traits that differentiate ecology, additional physiological traits that cannot be predicted from genomes, such as temperature optimum, substrate transport affinities, and biological interactions with phage and predators, may be relevant.

### Bacterial evolution in a changing Arctic Ocean.

The Arctic Ocean is a relatively enclosed ocean that is intensely influenced by riverine input. As such, Arctic Ocean surface waters represent a mosaic marine-freshwater interface that could be a global evolutionary hot spot for aquatic bacteria. Along with the introduction of vast amounts of freshwater and terrestrial nutrients (4, 6, 7), Arctic rivers may also serve as a conduit for the flow of organisms and genes from terrestrial to marine ecosystems. Indeed, a previous study showed that the ability of Arctic marine bacteria to use aromatic compounds of terrestrial origin evolved, at least in part, by lateral acquisition of genes from terrestrial bacteria (14). In the current study, we have shown that Arctic surface waters may support the evolution of bacteria that relatively recently colonized the oceans. Traditionally, these freshwater-marine transitions were considered rare in bacterial evolution (61). Some years ago, it was suggested that cross colonization was more common than currently realized and that previously undescribed invaders may be hiding in the “rare biosphere” (22). Indeed, a number of studies have since supported this hypothesis (50, 51), and BS01 certainly fits the criteria for rarity. Hence, the relatively fresh Arctic Ocean surface waters that are strongly influenced by terrestrial inputs may support a wider diversity of rare marine bacteria with relatively recent freshwater origins that are awaiting discovery. Finally, given the ongoing freshening of the Arctic Ocean, our results suggest that these relative newcomers to the ocean microbiome will increase in abundance and, therefore, ecological significance in a near-future Arctic Ocean.

## MATERIALS AND METHODS

### Sampling and metagenomic data generation.

Samples from 4 stations in the Canada Basin (CB2, CB4, CB8, and CB11) were collected aboard the CCGS *Louis S. St-Laurent* during the Joint Ocean Ice Study (JOIS) research mission in September 2015. Twelve samples were collected, and the associated environmental variables were measured for each sample (Table 1). Between 4 and 7 liters of seawater was sequentially filtered through a 50-μm-pore mesh, followed by a 3-μm-pore-size polycarbonate filter and a 0.22-μm-pore-size Sterivex filter (Durapore; Millipore, Billerica, MA, USA). Filters were preserved in RNAlater and stored at −80°C until processed in the laboratory. DNA was extracted from the Sterivex filters using an SDS lysis protocol as described in Colatriano et al. (14). DNA sequencing was performed at the Department of Energy Joint Genome Institute (Walnut Creek, CA, USA) on the HiSeq 2500-1TB (Illumina) platform using 150 PE technology.

### 16S rRNA gene and ITS analysis.

We used the complete 16S rRNA gene from OM43 strain HTCC2181 (19) to extract the 16S rRNA gene from each of the 12 single-sample metagenome assemblies using BLASTn (62). The sequences of  >500 bp were included in a phylogenetic analysis with reference sequences and 16S rRNA sequences from other biogeographic studies. A multiple-sequence alignment was generated using the MUSCLE algorithm as implemented in MEGA v.7 (63). A maximum likelihood tree was constructed using the GTR + gamma distribution (4 categories) model of nucleotide substitution in MEGA v.7 with 100 bootstraps (63).

We used the complete ITS region from a reference OM43 bacterium (HTCC2181) to extract ITS regions from each metagenome assembly using BLASTn (62). The sequences were clustered using CD-hit (64) at an identity of 100%. To assign sequences to specific clades, the Arctic ITS sequences were analyzed using reference sequences from published genomes and ITS sequences used in a previous biogeographic study (28). The sequences were aligned using the MUSCLE algorithm as implemented in MEGA v.7 (63), with poorly aligned sequences removed after visual inspection. A maximum likelihood tree was constructed using the GTR + gamma distribution (4 categories) model of nucleotide substitution in MEGA v.7 with 100 bootstraps (63). The distribution of subclades across the stations and depths was determined by summing the average read depth of all ITS sequences within each subclade. A principal coordinate analysis (PCoA) ordination of Bray-Curtis dissimilarities of the Arctic samples was performed based on the read depth of different ITS clades to determine the distribution across samples. The envdist function as implemented in vegan (65) with 999 permutations was used for post hoc tests of environmental variables.

### MAG generation and analysis.

Metagenomic binning was performed on scaffolds of >5 kb in length using MetaWatt (66). Binning was performed using tetranucleotide frequency, and the relative weight of coverage was set to 0.75, with the optimize bins and polish bins options on. The taxonomic identity of MAGs was assessed using a concatenated phylogenetic tree based on 138 single-copy conserved genes as implemented in MetaWatt (66). Estimation of MAG completeness and contamination was performed using CheckM (67), and suspected contamination was manually removed. A single putative BS01 MAG (Met-BS01-1) was identified from the CB2 surface metagenome for further analysis.

### Concatenated protein phylogeny.

The distribution of orthologous genes was analyzed using ProteinOrtho (68). Forty-eight single-copy orthologous genes present in all genomes were identified and selected for concatenated phylogenetic analysis. Each orthologous protein family was aligned using MUSCLE (implemented in MEGA6), and alignment positions were masked using the probabilistic masker ZORRO (69), masking columns with weights of <0.5. Phylogenetic reconstructions were conducted by maximum likelihood using MEGA6-v.0.6 and the following settings: JTT substitution model, gamma distribution with invariant sites model for the rate variation with four discrete gamma categories, and the nearest-neighbor interchange heuristic search method with a bootstrap analysis using 100 replicates.

### Comparative genomics.

Inference of protein function and metabolic reconstruction were based on the IMG annotations provided by the Department of Energy Joint Genome Institute (JGI) and using the Pathologic software available through Pathway Tools (70). The pangenomic visualization of *Methylophilaceae* was created using the anvi’o tool (71, 72). The toolbox of Rodriguez-R and Konstantinidi was used to compute the average amino acid identity (AAI) (73). The proteome isoelectric point was calculated with the software Pepstats from the EMBOSS package (74).

### Metatranscriptomic analysis.

RNA samples were collected during a JOIS mission in September 2017. RNA was extracted from the Sterivex filters (3- to 0.22-μm size fraction) with a modified protocol (75, 76), which employs both the mirVana miRNA isolation kit (Invitrogen) and the RNeasy RNA cleanup kit (Qiagen). cDNA library preparation and sequencing was performed at the JGI (Walnut Creek, CA) on the HiSeq 2500-1TB (Illumina) platform using 150 PE technology. To determine the activity and distribution of the Arctic *Methylophilaceae* MAG and reference genome HTCC2181 in the Arctic Ocean, unassembled metatranscriptomic data were recruited against the protein-coding gene sequences using BBMAP (https://jgi.doe.gov/data-and-tools/bbtools/bb-tools-user-guide/bbmap-guide/), with a minimum identity of 95%. The reads per kilobase of the MAG per gigabase of metatranscriptome (RPKG) were calculated to control for differences in raw reads between samples.

### Methanol dehydrogenase (XoxF4) phylogeny.

The full-length XoxF4 amino acid sequences from the reference isolate HTCC2181 and from the Met-BS01-1 MAG were used to assess the presence of BS01 in 1,362 metagenome assemblies from aquatic communities available at IMG/M using BLASTp (62). The sequences of >300 bp were included in a phylogenetic analysis with reference sequences and sequences from other biogeographic studies. A multiple-sequence alignment was generated using the MUSCLE algorithm as implemented in MEGA v.7 (63). A maximum likelihood tree was constructed using the JTT substitution model, gamma distribution (4 categories), and the nearest-neighbor interchange heuristic search method in MEGA v.7 with 100 bootstraps (63).

### Fragment recruitment.

The distribution of the Arctic *Methylophilaceae* MAG and HTCC2181 in the multiple aquatic biomes was determined using the best-hit reciprocal blast approach reported in Colatriano et al. (14). Unassembled metagenomics data from 45 samples at 20 sites (Table S2) were recruited to the Arctic *Methylophilaceae* MAG and HTCC2181. All hits from the initial blast were then reciprocally queried against the Arctic *Methylophilaceae* MAG and HTCC2181. The best hit was reported, and hits with an alignment length of  ≥100 bp and a percent identity of  ≥95% were counted. To compare the results among the different data sets, the number of recruited reads was normalized to the total number of reads in each sample. The final coverage results were expressed as the number of RPKG.

10.1128/mBio.01306-21.6 TABLE S2 List of aquatic metagenomes used in the fragment recruitment analysis. Download Table S2, XLSX file, 0.01 MB. Copyright © 2021 Ramachandran et al. 2021 Ramachandran et al. https://creativecommons.org/licenses/by/4.0/ This content is distributed under the terms of the Creative Commons Attribution 4.0 International license.

### *xoxF4* primer design and qPCR.

Primer sets were designed to amplify an ∼200-bp fragment of the *xoxF4* gene from BS01 or OM43 specifically. The primer set specific to BS01 was F1024_BS01 (5′-ATT GCT AAA TGG GGC TAC-3′) and R1161_BS01 (5′-GTT GAA TGT ATA TGC GAA ACC-3′). The primer set specific to OM43 was F1015_OM43 (5′-GAY TTA GAY ACA GGT ATG GCR-3′) and R1161_OM43 (5′-CCA TGT GTA WGC AAA ACC GTT TCT-3′). Specificity of primer sets was validated through cloning and sequencing of the DNA inserts (Fig. S4). Cloning, cDNA synthesis, PCR, and qPCR were performed as described in Ramachandran and Walsh (24), with the annealing temperature of 52.3°C for both primer sets.

10.1128/mBio.01306-21.4 FIG S4 Validation of the specificity of the BS01 and OM43 PCR primer sets via sequencing of cloned PCR products and phylogenetic analysis. The tree was inferred using maximum likelihood (500 bootstraps) and a GTR + gamma distribution (four categories) with invariants sites model of evolution using nearest-neighbor interchange heuristic search method. Boldface taxa are the sequences recovered by either primer set. Download FIG S4, EPS file, 0.8 MB. Copyright © 2021 Ramachandran et al. 2021 Ramachandran et al. https://creativecommons.org/licenses/by/4.0/ This content is distributed under the terms of the Creative Commons Attribution 4.0 International license.

### Data availability.

The metagenomic data were deposited in the IMG database under GOLD Project IDs Gp0134345 to Gp0134356. The metatranscriptome data are deposited in the IMG database under GOLD Project IDs Gp0323995, Gp0324000, and Gp0323990.
