# Supplementary material for: A Novel Freshwater to Marine Evolutionary Transition Revealed within Methylophilaceae Bacteria from the Arctic Ocean
Source: mBio. 2021 Jun 22;12(3):e01306-21. doi: 10.1128/mBio.01306-21 (PMC8262872; doi:10.1128/mBio.01306-21)
Supplement: TABLE S1 [file mbio.01306-21-st001.pdf]

Table S1. Characteristics of Methylophilaceae genomes

	Met-BS01-1	HTCC2181	KB13	MBRS-H7	'Ca. M. rimovensis' MMS-RI-1	'Ca. M. planktonicus' MMS-2-53	'Ca. M. universalis' MMS-VB-103	'Ca. M. turicensis' MMS-10A-171	Methylotenera versatilis 79	Methylotenera mobilis 13	Methylobacillus flagellatus KT
Isolation location	Beaufort Sea	Oregon	Hawaii	Red Sea	Římov reservoir	Zurich	Zurich	Zurich	Washington	Washington	-
Isolation source	Seawater	Seawater	Seawater	Seawater	Lake	Lake	Lake	Lake	Lake sediment	Lake sediment	Activated sludge
Genome size (Mbp)	1.5	1.3	1.3	1.4	1.3	1.4	1.3	1.8	2.6	2.8	3.0
GC content (%)	42.7	37.9	35.3	35.5	36.7	37.0	37.0	44.5	41.9	45.6	55.7
AAI with Met-BS01-1 (%)	-	56	53	54	59	59	59	64	69	66	62
Lysine content (%)	6.0	7.5	8.1	8.1	7.8	7.8	7.9	5.7	5.8	5.6	4.2
Arginine content (%)	4.4	3.9	3.5	3.6	4.0	4.0	4.0	4.7	4.3	4.7	5.9
